# Right ventricular function and its relationship with grade of hepatosteatosis in non-alcoholic fatty liver disease

**DOI:** 10.5830/CVJA-2014-068

**Published:** 2015

**Authors:** Adem Bekler, Emıne Gazi, Gokhan Erbag, Emine Binnetoglu, Ahmet Barutcu, Hacer Sen, Ahmet Temiz, Burak Altun

**Affiliations:** Department of Internal Medicine, Canakkale Onsekiz Mart University, Çanakkale, Turkey; Department of Internal Medicine, Canakkale Onsekiz Mart University, Çanakkale, Turkey; Department of Internal Medicine, Canakkale Onsekiz Mart University, Çanakkale, Turkey; Department of Internal Medicine, Canakkale Onsekiz Mart University, Çanakkale, Turkey; Department of Internal Medicine, Canakkale Onsekiz Mart University, Çanakkale, Turkey; Department of Internal Medicine, Canakkale Onsekiz Mart University, Çanakkale, Turkey; Department of Internal Medicine, Canakkale Onsekiz Mart University, Çanakkale, Turkey; Department of Internal Medicine, Canakkale Onsekiz Mart University, Çanakkale, Turkey

**Keywords:** echocardiography, hepatosteatosis, right ventricular dysfunction

## Abstract

**Objective:**

This study was designed to assess right ventricular systolic and diastolic function and its relationship with grade of hepatosteatosis (HS) in non-alcoholic fatty liver disease (NAFLD) patients using conventional and tissue Doppler echocardiography.

**Methods:**

NAFLD was diagnosed in 32 individuals (15 males, 17 females; 59% were grade I HS, 41% grade II–III HS) by means of ultrasonography. Twenty-two individuals, whose ultrasonography data did not show HS, comprised the control group (11 males, 11 females) and were included in the study. Right ventricular systolic and diastolic function and their relationship with grade of HS were assessed by conventional and tissue Doppler echocardiography. Additionally, right ventricular global function was assessed by myocardial performance index (MPI).

**Results:**

When compared by conventional echocardiographic parameters, there were no significant differences between the two groups. With tissue Doppler parameters, the tricuspid annulus peak early diastolic velocity and ratio of early-tolate diastolic velocity were lower in the patients than in the controls (*p* = 0.03, *p* = 0.02, respectively). The isovolumetric relaxation time and MPI were significantly higher (*p* < 0.001, *p* < 0.001, respectively) in the patient group. HS grade was positively correlated with right ventricular isovolumetric relaxation time and MPI index (*r* = 0.295, *p* = 0.03, *r* = 0.641, *p* < 0.001, respectively).

**Conclusion:**

These results show that right ventricular diastolic dysfunction (RVDD) in patients with NAFLD and degree of HS was associated with RVDD.

## Abstract

Non-alcoholic fatty liver disease (NAFLD) is increasingly recognised as the most common cause of chronic liver disease worldwide.^1^ NAFLD encompasses a spectrum of disorders, including variable degrees of simple hepatic steatosis (HS, fatty liver), non-alcoholic steatohepatitis (NASH) and cirrhosis.

This disease is a common clinicopathological condition characterised by significant lipid deposition in the hepatocytes of the liver parenchyma in the absence of alcohol abuse, contributing medications and viral hepatitis. It is strongly associated with several cardiovascular risk factors such as obesity, insulin resistance, hypertension, hyperlipidaemia, coronary artery disease, obstructive sleep apnoea syndrome, oxidative stress, endothelial dysfunction and the metabolic syndrome.^2^-^5^ There are recent data suggesting that NAFLD is linked to increased cardiovascular risk independent from the broad spectrum of metabolic syndrome (MS) risk factors.^6^,^7^

Multiple mechanisms contribute to left ventricular dysfunction in obesity, including lipotoxicity associated with cardiac steatosis and lipo-apoptosis, alterations in fatty acid metabolism, overproduction of cardio-inhibitory cytokines, up-regulation of some neurohormones (especially angiotensin II), myocardial fibrosis and chronic overload with left ventricular dilatation and hypertrophy, and increased oxygen consumption.^8^

Evaluating the possible influence and correlation of metabolic, cardiovascular and liver biopsy parameters on cardiac left ventricular dysfunction, we found a positive correlation between left ventricular parameters and severity of liver damage (NAS score).^9^ Cardiac dysfunction determined by echocardiographic measurements in patients with NAFLD was also studied.^10^ Determination of myocardial velocity using tissue Doppler imaging (TDI) is a new technique that has recently been developed to analyse right ventricular function.^11^-^14^

This study aimed to investigate the association between right ventricular function and grade of hepatosteatosis (HS grade) in NAFLD patients using transthoracic and tissue Doppler echocardiography.

## Methods

Thirty-two patients, who were admitted to the Internal Medicine Clinic at our institution between 2011 and 2012 and were diagnosed with hepatosteatosis using abdominal ultrasonography (USG), performed for any reason, were included in the study, taking into account the exclusion criteria. Twenty-two persons were also included in the study as a control group. To eradicate the effects of other variables on the impact of NAFLD on right ventricular function, the control group was selected according to the demographic and laboratory characteristics of the patients with NAFLD. Each participant signed an informed consent form in accordance with the Declaration of Helsinki, and this study was approved by the local ethics committee of our hospital.

The exclusion criteria were as follows: previous coronary artery disease (patients who had a history of myocardial infarction, unstable angina pectoris, angiographically proven significant coronary artery stenosis or had undergone revascularisation), congestive heart failure (left ventricular ejection fraction ≤ 40% or symptomatic heart failure), patients who had known or a history of valvular heart disease, pulmonary disease, pulmonary hypertension, left bundle branch block, a rhythm other than sinus, and pericarditis. Chronic alcohol consumption (more than 20 g/day), serum hepatitis B antigen or anti-hepatitis C viral antibody positivity, which are known to worsen NAFLD, were the other exclusion criteria.

All medications were stopped 48 hours before the time of echocardiography. Fasting venous blood samples were taken to determine levels of blood glucose, electrolytes, total cholesterol, high-density lipoprotein cholesterol, low-density lipoprotein cholesterol and triglycerides.

## Ultrasonography

Although liver biopsy is currently the gold standard for distinguishing NAFLD forms, abdominal USG is the preferrred method for qualitative assessment of NAFLD.^10^ Abdominal USG was performed on all study participants by a single experienced physician who was blinded to the clinical and laboratory results of the study groups.

The diagnosis of NAFLD was based on increased liver echotexture on ultrasonography [Siemens Antares (Erlangen, Germany)] compared with the kidneys, vascular blurring and deep attenuation.^15^ Fat infiltration in the liver was described in three ultrasonographic stages using published criteria.^16^,^17^ The liver was considered to be normal if there was normal hepatic echotexture and normal beam attenuation.

Mild steatosis (grade I) was identified as a minimal increase in echogenicity of the liver parenchyma with a slight decrease in definition of the portal vein walls and minimal or no posterior beam attenuation. Severe steatosis (grade III) was identified as grossly increased hepatic parenchymal echotexture that permitted visualisation of the main portal vein walls alone. Smaller venules were not visualised, and there was increased posterior beam attenuation. Moderate steatosis (grade II) was identified by hepatic echogenicity, portal venous definition and beam attenuation between mild and severe parameters. According to USG results, 59% grade I HS and 41% grade II–III HS was found in the patients.

## Echocardiography

All patients underwent a complete transthoracic echocardiographic and tissue Doppler study using multiple views in the left lateral decubitus position. Echocardiographic measurements were calculated by two of three experienced cardiologists who were blinded to the current study. In case of disagreement, an opnion was obtained from the third cardiologist, and the final decision was made by consensus.

This study was performed using a 3.5-Mhz transducer on a Vivid 7 GE ultrasonographic system. Echocardiographic measurements were made in accordance with the criteria recommended by the American Society of Echocardiography. All subjects were in sinus rhythm. The measurements were done on three consecutive heartbeats, and the average of these measurements was calculated.

In the apical four-chamber view, the sample volume (size 2 mm) of the pulsed-wave Doppler was placed between the tips of the tricuspid leaflets. The tricuspid inflow velocity was traced and the following variables were measured: peak velocity of early (E) and late (A) filling and deceleration time (DT) of the E-wave velocity.

In the parasternal long-axis view, the right ventricular (RV) diameter was measured using M mode from the RV anterior wall to the right side of the interventricular septum on the R wave of the electrocardiogram. RV longitudinal functions were assessed by pulsed tissue Doppler imaging (TDI). Pulsed Doppler sample volume (size 5 mm) was placed on the basal portion of the right ventricle at the level of the lateral tricuspid annulus from the apical four-chamber view. The Nyquist limit was set at 15 to 20 cm/s. For optimising the spectral display of myocardial velocities, the monitor sweep speed was adjusted at 50 to 100 mm/s.

The pulsed TDI pattern has a positive myocardial systolic velocity (Sa) and two negative diastolic velocities: early (Ea) and late (Aa). The diastolic indices of myocardial early (Ea) and atrial contraction (Aa) peak velocities and myocardial systolic wave (Sa) velocity were measured and the ratio of Em/Am was calculated. TDI-derived myocardial performance index (MPI) of the right ventricle was measured by dividing the difference between the time interval from the end to the onset of the tricuspid annular velocity pattern during diastole (*a*) and the duration of the tricuspid Sa (*b*) by the tricuspid Sa duration (*b*).

RV MPI ((a-b))/b

Conventional and tissue Doppler echocardiographic parameters and their implications on right ventricular systolic and diastolic function are presented in Table 1.

**Table 1:** Conventional and tissue Doppler echocardiographic parameters and their implications on right ventricular systolic and diastolic function

*Parameters*	*Systolic function*	*Diastolic function*
Conventional echocardiography		
Tricuspid E	–	+
Tricuspid Aa	–	+
Tricuspid E/A	–	+
Tricuspid E DT	–	+
Tissue Doppler imaging		
RV S′	+	–
RV Ea′	–	+
RV Aa′	–	+
RV Ea′/Aa′	–	+
RV deceleration time (ms)	–	+
Isovolumetric relaxation time (ms)	–	+
Isovolumetric contraction time (ms)	+	–
Contraction time (ms)	+	–
Myocardial performance index	+	+

Tricuspid E: peak velocity of early diastolic filling, tricuspid A: peak velocity of atrial diastolic filling, tricuspid E DT: deceleration time of early diastolic filling, Ea: tricuspid lateral annulus early diastolic wave, Aa: tricuspid lateral annulus late diastolic wave, DT: tricuspid lateral annulus E-wave deceleration time, RV: right ventricular.

## Biochemical evaluation

Blood samples were drawn from each patient after a 12-hour overnight fast for the determination of lipid profiles and glucose levels. Plasma glucose level was determined with the glucose oxidase/peroxidase method (Gordion Diagnostic, Ankara, Turkey). Levels of total cholesterol, high-density lipoprotein cholesterol (HDL-C), and triglycerides (TG) were determined with enzymatic colorimetric assays by spectrophotometry. Low-density lipoprotein cholesterol (LDL-C) was calculated using the Friedewald formula.

## Statistical analysis

The SPPS version 20.0 software package was used for statistical analysis. All the data were expressed as mean ± standard deviation. The Kolmogorov–Smirnov test was used to determine normal disributions. Categorical variables were compared with the chi-square or Fisher’s exact test. Normally distributed variables were compared across groups by means of the Student’s *t*-test whereas variables that did not normally distribute were compared by means of the Mann–Whitney *U*-test. Spearman’s correlation analysis was used to evaluate the relationship between the variables. A *p*-value < 0.05 was considered significant.

## Results

NAFLD was diagnosed in 32 individuals (15 males, 17 females; mean age 50 ± 9 years; 59% with grade I HS, 41% grade II–III HS) by means of ultrasonography data. Twenty-two individuals whose ultrasonography data did not show HS comprised the control group (11 males, 11 females; mean age 50 ± 10 years) and were included in the study.

Clinical characteristics of the 32 NAFLD patients and 22 control subjects are presented in Table 2. Age, gender, body mass index, diabetes mellitus, dyslipidaemia and smoking status were similar between the NAFLD patients and controls. Additionally, there were no significant differences with regard to laboratory results (Table 2). When compared in terms of echocardiographic features, the groups were similar in chamber diameter and standard Doppler parameters (Table 3).

**Table 2:** Demographic and clinical characteristics of the groups

*Parameters*	*Patient group (n = 32)*	*Control group (n = 22)*	*p-value*
Mean age (years)	50 ± 9	51 ± 10	0.979
Gender M/F	15/17	11/11	0.821
Body mass index (kg/m²)	29.8 ± 3.4	28.2 ± 3.2	0.094
Smoking, n (%)	5 (15)	7 (31)	0.160
Hypertension, n (%)	6 (18)	6 (27)	0.459
Diabetes mellitus n (%)	4 (12)	3 (13)	0.903
Dyslipidaemia, n (%)	11 (34)	5 (22)	0.357
Glucose (mg/dl)	105.4 ± 21.4	102.8 ± 28.9	
(mmol/l)	5.85 ± 1.19	5.71 ± 1.60	0.174
AST (U/l)	25.7 ± 6.9	25.2 ± 7.4	0.711
ALT (U/l)	28.5 ± 12.1	26.1 ± 12.2	0.459
LDH (U/l)	159.5 ± 21.7	156.3 ± 17.8	0.672
Haemoglobin (g/dl)	14.1 ± 1.1	13.8 ± 1.3	0.622
Total cholesterol (mg/dl)	200.7 ± 40.3	184.3 ± 40.3	0.119
(mmol/l)	5.20 ± 1.04	4.77 ± 1.04	
Triglyceride (mg/dl)	184 ± 85.5	158.4 ± 77.9	0.189
(mmol/l)	2.08 ± 0.97	1.79 ± 0.88	
High-density lipoprotein (mg/dl)	40 ± 8.8	39.5 ± 11.2	0.433
(mmol/l)	1.04 ± 0.23	1.02 ± 0.29	
Low-density lipoprotein (mg/dl)	124.7 ± 35	113.4 ± 35.8	0.144
(mmol/l)	3.23 ± 0.91	2.94 ± 0.93	

AST: aspartate aminotransferase, ALT: alanine transaminase, LDH: lactate dehydrogenase.

**Table 3:** Echocardiographic parameters between the patient group and the control group

*Parameters*	*Patient group*	*Control group*	*p-value*
LVEDD (mm)	48.07 ± 3.82	48.47 ± 3.95	0.986
LVESD (mm)	29.8 ± 4.78	30.43 ± 4.22	0.140
IVS (mm)	10.08 ± 1.33	9.90 ± 1.19	0.720
LVEF (%)	68.01 ± 7.01	66.31 ± 6.59	0.762
RVEDD (mm)	28.78 ± 3.15	28.47 ± 3.67	0.523
LA diameter (mm)	36.35 ± 3.81	36.94 ± 3.08	0.710
RA diameter (mm)	32.99 ± 4.93	31.31 ± 4.72	0.535
Tricuspid E (cm/s)	55.8 ± 12.6	54.6 ± 8.3	0.888
Tricuspid A (cm/s)	47 ± 13	41.7 ± 9.6	0.156
Tricuspid E/A ratio	1.2 ± 0.4	1.3 ± 0.3	0.413
Tricuspid E DT (ms)	190.2 ± 30.4	193.6 ± 19.5	0.365

LVEDD: left ventricular end-diastolic diameter, LVESD: left ventricular end-systolic diameter, IVS: interventricular septum, LVEF: left ventricular ejection fraction, RVEDD: right ventricular end-diastolic diameter, LA: left atrium, RA: right atrium, tricuspid E: peak velocity of early diastolic filling, tricuspid A: peak velocity of atrial diastolic filling, tricuspid E DT: deceleration time of early diastolic filling.

There were no significant differences for E, A, E/A and DT between the patients and controls. For tissue Doppler parameters, the Ea and Ea/Aa were lower in the patient group than in the control group (*p* < 0.001, *p* < 0.001, respectively). Isovolumetric relaxation time (IVRT) and MPI were significantly higher (*p* = 0.002, *p* < 0.001, respectively) in the patient group. There were no significant differences for Aa, DTa, Sa, isovolumetric contraction time (IVCT) and contraction time (CT) between the two groups. Tissue Doppler parameters are presented in Table 4.

**Table 4:** Right ventricular tissue Doppler echocardiographic parameters

*Parameters*	*Patient group*	*Control group*	*p-value*
RV S-wave peak velocity (cm/s)	13.8 ± 2.1	14.3 ± 2.1	0.476
RV Ea-wave peak velocity (cm/s)	12.1 ± 3.1	14 ± 3.1	0.03
RV Aa-wave peak velocity (cm/s)	17.2 ± 3.6	15.5 ± 3.9	0.061
RV Ea/Aa ratio	0.7 ± 0.3	0.9 ± 0.4	0.02
Ea DT (ms)	166.2 ± 27.7	167.2 ± 22.7	0.679
IVRT (ms)	77.3 ± 8.5	61.5 ± 4.3	< 0.001
IVCT (ms)	66.4 ± 8.7	62.8 ± 9.9	0.072
CT (ms)	295.5 ± 27.6	302.1 ± 23 5	0.610
Tei index LV	0.50 ± 0.05	0.42 ± 0.03	< 0.001

RV: right ventricular, Ea: tricuspid lateral annulus early diastolic wave, Aa: tricuspid lateral annulus late diastolic wave, DT: tricuspid lateral annulus E-wave deceleration time, IVRT: isovolumetric relaxation time, IVCT: isovolumetric contraction time, CT: contraction time.

Grade of HS was positively correlated with right ventricular isovolumetric relaxation time and MPI (*r* = 0.295, *p* = 0.03, *r* = 0.641, *p* < 0.001, respectively). Correlations between grade of HS and echocardiographic parameters are shown in Table 5.

**Table 5:** Correlations between grade of hepatosteatosis and echocardiographic parameters

*Parameters*	*r*	*p-value*
Ea	0.020	0.886
Ea/Aa	–0.156	0.260
IVRT*	0.295	0.03
MPI*	0.641	< 0.001

*By hepatosteatosis, IVRT: isovolumetric relaxation time, MPI: right ventricular myocardial performance index, HS: hepatosteatosis.

## Discussion

The results of this study indicate that the presence of NAFLD was associated with impaired RV diastolic function (RVDF). In addition, the evidence showed that the existence of NAFLD was related to the extent of impairment in RVDF. NAFLD is more common in patients with impaired RVDF, obesity, insulin resistance, hypertension, hyperlipidaemia, coronary artery disease, obstructive sleep apnoea syndrome, oxidative stress, endothelial dysfunction and the metabolic syndrome.

In this study, although RVDF was impaired, systolic function was preserved in NAFLD patients. Furthermore, HS grade was positively correlated with RV MPI. Several studies reported only diastolic left ventricular dysfunction, while others reported both diastolic and systolic left ventricular functional impairment in NAFLD patients with hypertension, insulin resistance, type 2 diabetes and/or the metabolic syndrome.^18^-^20^

Evaluating the possible influence and correlation of metabolic, cardiovascular and liver biopsy parameters on cardiac left ventricular dysfunction, we found a positive correlation between left ventricular parameters and severity of liver damage (NAS score).^9^ However, to the best of our knowledge, to date, no study has explored the involvement of right ventricular systolic and diastolic function and its relationship with HS grade in NAFLD patients.

We speculated that the excessive lipid accumulation in hepatocytes that is a characteristic of NAFLD can lead to lipid deposition in cardiac myocytes, promoting RVDF. In addition, our study showed that significantly impaired RVDF was associated with HS grade in NAFLD according to TDI. However, we could not detect any significant difference in tricuspid lateral annulus systolic velocity between the NAFLD patients and controls. Therefore, right ventricular systolic function was not impaired in patients with NAFLD.

MPI is a DTI-derived quantitative parameter used frequently in recent years to grade systolic and diastolic function. This index was first described by Tei *et al.*^21^ and is widely accepted because it correlates with more invasive measurements, is easy to reproduce and is easy to perform. In coronary artery disease, a prolonged MPI is an important disease precursor observed before the development of systolic dysfunction.^22^ A markedly prolonged MPI despite an unchanged tricuspid lateral annulus systolic velocity in NAFLD patients compared with the control group is compatible with the hypothesis that prolonged MPI stems from RVDD. In fact, RVDF deteriorates in NAFLD, and this deterioration is associated with grade of HS.

There were some limitations to our study. The first was the small sample size. The second was that the diagnostic method depended on USG, and the exclusion of other secondary causes of chronic liver disease was not confirmed by liver biopsy. Although liver biopsy is currently the gold standard for distinguishing NAFLD forms, for assessing the severity of damage and prognosis, NAFLD can be detected as a bright liver on USG, which is possible to perform routinely. Moreover, liver USG has proven to be a sensitive, accurate and convenient diagnostic tool in detecting steatosis. Its sensitivity ranges from 60 to 94% and its specificity from 84 to 95%.^15^,^23^

## Conclusion

We found that there was significant impairment in diastolic function in non-diabetic and normotensive NAFLD patients compared to the controls. It should be kept in mind that diastolic function may be impaired while systolic function is preserved in NAFLD patients. We suggest that patients with NAFLD require aggressive cardiac risk-factor modification and closer follow up for the prevention of diastolic and systolic heart failure.
